# Hippocampal volume loss following childhood convulsive status epilepticus is not limited to prolonged febrile seizures

**DOI:** 10.1111/epi.12426

**Published:** 2013-10-28

**Authors:** Michael Yoong, Marina M Martinos, Richard F Chin, Christopher A Clark, Rodney C Scott

**Affiliations:** * Neurosciences Unit, UCL Institute of Child HealthLondon, United Kingdom; † Imaging and Biophysics Unit, UCL Institute of Child HealthLondon, United Kingdom; ‡ Young EpilepsyLingfield, Surrey, United Kingdom; § Developmental Cognitive Neurosciences Unit, UCL Institute of Child HealthLondon, United Kingdom; ¶ Edinburgh Neurosciences, Muir Maxwell Epilepsy Centre, The University of EdinburghEdinburgh, United Kingdom; # Department of Neurology, Geisel School of Medicine at DartmouthLebanon, New Hampshire, U.S.A

**Keywords:** Status epilepticus, Mesial temporal sclerosis, Prolonged febrile seizures, Epilepsy, Hippocampus

## Abstract

**Purpose:**

Childhood convulsive status epilepticus (CSE), in particular prolonged febrile seizures (PFS), has been linked with mesial temporal sclerosis (MTS). Previous studies have shown that hippocampal injury occurs in the acute phase immediately following CSE, but little is known about the longer term evolution of such injury. This study aimed to investigate the longer term outcome of childhood CSE with sequential magnetic resonance imaging (MRI) looking for progressive hippocampal injury during the first year post-CSE.

**Methods:**

Eighty children (0.18–15.5 years) underwent brain MRI 1 month post-CSE, 50 with a repeat MRI at 6 months and 46 with repeat MRI at 12 months post-CSE. Thirty-one control subjects without neurologic problems had a single brain MRI for comparison. Hippocampal volumes were measured from each MRI scan by two independent observers, and hippocampal growth rates were estimated in each patient with suitable imaging.

**Key Findings:**

Hippocampal volume loss was found in 20–30% of patients and was not associated with the etiology or clinical features of CSE, including seizure duration or focality. A borderline association was found between a history of previous seizures (p = 0.063) and the number of previous febrile seizures (p = 0.051), suggesting that multiple insults may be important in the pathogenesis of progressive hippocampal injury.

**Significance:**

It is apparent that progressive hippocampal damage can occur after CSE of any etiology and is not limited to PFS. Repeated seizures may play an important role, but further follow-up is needed to determine any other risk factors and proportion of children showing initial volume loss progress to clinical MTS and temporal lobe epilepsy.

Prolonged seizures have long been linked with mesial temporal sclerosis (MTS), the most common pathology seen in patients with temporal lobe epilepsy (TLE; Grattan-Smith et al., 1993). Around 30–50% of patients with intractable TLE secondary to MTS have had an episode of childhood convulsive status epilepticus (CSE; Cavanagh & Meyer, 1956; Harvey et al., 1995), and childhood prolonged febrile seizures (PFS) in particular, where CSE occurs in a neurologically normal child during a febrile illness, have been specifically identified as a potential causative factor for MTS (Cascino, 1995; Maher & McLachlan, 1995).

Case reports have suggested that prolonged seizure activity can be associated with brain injury, including progressive hippocampal damage, ultimately leading to MTS (Pohlmann-Eden, 2004). CSE is a relatively common condition in children, with an incidence of 17–23/100,000 children per year (Chin et al., 2006); hence this has potentially serious implications for medical care. It is therefore important to systematically address this issue to assess the risks.

Studies performing magnetic resonance imaging (MRI) in children in the first few days following PFS have consistently shown acute increases in hippocampal volume and hyperintensities on T_2_-weighted sequences (VanLandingham et al., 1998; Scott et al., 2002; Natsume et al., 2007; Shinnar et al., 2012). These are interpreted as representing acute hippocampal edema. The longer term evolution of these acute changes is less well characterized, and in particular how or whether they are associated with MTS remains unclear (Tarkka et al., 2003).

Follow-up imaging of children with PFS has shown that initial increases in hippocampal volume and T_2_ relaxation time resolve over time (VanLandingham et al., 1998; Scott et al., 2003; Provenzale et al., 2008), but that increased hippocampal asymmetry (Scott et al., 2003; Farrow et al., 2006) or unilateral volume loss (Provenzale et al., 2008) may occur in some children. The present study was designed to prospectively investigate hippocampal changes in a new cohort of children with CSE over the initial year post-CSE and estimate the effect of CSE on subsequent hippocampal growth. Investigations were targeted to characterize hippocampal growth from 1 month to 1 year following the episode of CSE and avoiding the period immediately following CSE to prevent confounding from the previously demonstrated acute changes. We hypothesized that by repeating hippocampal measurements at multiple time points over the year, evolving loss of hippocampal volume prior to the development of clinical MTS and TLE would be detected. A secondary hypothesis was that this would be specific to children with PFS and not those with other forms of CSE.

## Materials and Methods

Between 2007 and 2010, a cohort of 80 children was recruited from hospitals in the North Thames health region, London, United Kingdom, following an episode of CSE (defined as a convulsive seizure or series of seizures lasting longer than 30 min without recovery of consciousness). The study was approved by the Great Ormond Street Hospital (GOSH) research ethics committee.

Enrolled children were seen at GOSH for an assessment, including detailed clinical review and MRI investigations 1 month after the acute episode. MRI investigations were performed with the child awake, in natural sleep, under sedation, or under general anaesthesia as appropriate for the age and developmental stage of the child. These investigations were repeated at 6 and 12 months after the initial episode of CSE.

Patients were divided into PFS and non-PFS groups for analysis, where PFS was defined as an episode of CSE occurring in a previously neurologically healthy child, associated with a fever >38°C, in the absence of defined central nervous system infection.

Twelve healthy volunteers and 19 children having MRI scans for other clinical reasons, including dermatologic or ophthalmic lesions, with no previous history of seizures and no reported developmental or neurologic problems, were recruited as controls. All volunteers were scanned either awake or during natural sleep. Children who showed intracranial lesions on their radiologic report were excluded from the control group. Unlike children with CSE, who had repeated assessments, each control only had a single MRI investigation as: (1) repeating the general anesthesia or sedation for those children who were having MRI for clinical reasons would not have been ethical and (2) repeating an MRI scan on those young controls that had their MRI during postprandial natural sleep 1 year later would have required sedation. All MRI investigations were performed on the same 1.5-T scanner (Avanto; Siemens, Erlangen, Germany) using a protocol including a T_1_-weighted three-dimensional Fast Low Angle Shot (3D-FLASH) sequence (repetition time (TR) = 4.94 msec, echo time (TE) = 11 msec, acquisition matrix 256 × 224, in-plane resolution 1.0 × 1.0 mm, slice thickness 1 mm).

### Hippocampal and intracranial volume measurement

Quantitative measurement of hippocampal volumes was performed using the images obtained from 3D-FLASH sequences. Using MRIcroN (http://www.mccauslandcenter.sc.edu/mricro/mricron/index.html), regions of interest (ROIs) were drawn manually on successive coronal slices, using axial and sagittal views for further refinement, to encompass the entire hippocampus. The anatomic limits of the hippocampus were defined using the description given by Gousias et al. (2008; Hammers et al., 2003). This was performed separately by two individual observers (MY and MM), blinded to the clinical status of the child, and measurements were averaged for analysis.

The *brain extraction tool* (BET) in FSL (Analysis Group, FMRIB, Oxford, United Kindgom) was used to segment brain from skull and overlying tissue and to calculate intracranial volume (ICV). Each image was manually inspected by one researcher (MY) and manually adjusted to minimize segmentation errors.

### Statistics

All statistical analyses were performed with SPSS for Windows version 19.0 (Chicago, IL, U.S.A.). p < 0.05 was taken as the cutoff for significance, and Bonferroni corrections for multiple comparisons were made where appropriate. Reliability of hippocampal measurements was assessed using averaged measurements from each individual observer to calculate the coefficient of variation (CoV) for intra-rater and interrater measurements.

Cross-sectional comparison between the control group and patients at each time point was performed using univariate analysis of variance (ANOVA) with ICV as a covariate. The same control group was used for each comparison.

Because for ethical and practical reasons longitudinal measurements were not available on control children, it was not possible to directly compare growth rates in patients and controls. In patients with CSE, linear regression was used to estimate left and right hippocampal growth rates over the study period for those who had one or more follow-up scans. The 95% confidence interval (CI) of this estimate was calculated, and patients with the upper limit of this CI below 0, were considered to have “definite” loss of hippocampal volume. The control sample was modeled using a negative decay curve in order to confirm that on a group basis hippocampi should increase in size over the age range of our patients. Therefore, we have taken a conservative approach to inference by only considering patients with a CI for growth of <0 as having impairment in hippocampal growth.

Linear and logistic regression was used to explore the influence of baseline clinical factors on hippocampal growth.

## Results

### Demographics

A total of 225 children with CSE were identified as eligible to take part in the study, 80 of whom were enrolled and underwent initial MRI investigations. Of those who were not enrolled, 35 children could not be contacted owing to missing or incorrect contact details; 38 children were unsuitable for MRI under sedation owing to instability of their clinical condition/comorbidities; 48 children declined to participate; 15 children who lived distant to the study area were not willing to visit our center; and 7 children died during their acute hospital admission. An additional two children agreed to participate but did not attend their appointments. Because these children were not seen for assessment and so did not consent to participate in the study, only minimal demographic details were available, and a comparison of clinical features of these children and those enrolled was not possible. They did not differ significantly from participants in age (mean age 3.8 vs. 3.2 years; *t*-test; p = 0.175) or sex (male-to-female ratio 39:41 vs. 85:60; chi-square, p = 0.154). There was a difference in etiologic composition, with children acute symptomatic CSE forming 15.8% of those referred, but only 3.5% of those enrolled (chi-square, p < 0.001).

There were 33 children with PFS and 47 with non-PFS CSE. These comprised 22 patients with symptomatic epilepsy, 21 with idiopathic/genetic epilepsy, and 4 who presented with a first episode of afebrile CSE with no previous seizures. Their demographic and clinical details are summarized in Table 1. Children with non-PFS were more likely than children with PFS to have had focal CSE (chi-square, p = 0.046), previous seizures (chi-square, p < 0.001), or a previous episode of CSE (chi-square, p = 0.001). There were no statistically significant between-group differences in median age or seizure duration (Mann-Whitney *U*, p = 0.077 and p = 0.652, respectively). There were no significant differences between patients who attended one, two, or three scans with respect to age, seizure duration, or any other clinical factors. The numbers of patients available at each follow-up are summarized in Table 2.

**Table 1:** Demographics and seizure characteristics of cohort

	PFC (n = 33)	Non-PFS CSE (n = 47)	Overall (n = 80)	Controls (n = 31)
Median age/years (range)	1.86 (0.80–4.61)	2.40 (0.18–15.50)	2.28 (0.18–15.50)	3.03 (0.21–12.69)
Male-to-female ratio	10:23	29:18	39:41	12:19
Median time to first scan/days (range)	37 (5–90)	22 (7–66)	29.5 (5–90)	
Mean time to first scan/days (SD)	37.8 (20.2)	27.5 (15.6)	31.8 (18.2)	
Focal onset (%)	5 (15.2)	18 (38.3)	23 (29.1)	
Continuous seizure activity (%)	20 (61)	26 (55)	46 (57)	
Intermittent seizure activity (%)	13 (39)	21 (45)	54 (43)	
Mean seizure duration in minutes (range)	71.67 (30–190)	92.9 (30–265)	72.40 (30–265)	
Previous seizures (%)	12 (36)	36 (77)	48 (60)	
Previous episode CSE (%)	2 (6)	19 (40)	21 (26)	

**Table 2:** Patient numbers at each follow-up assessment

	PFS	Non-PFS	Overall
1st MRI scan	33	47	80
2nd MRI scan	21	29	50
3rd MRI scan	21	25	46

#### Hippocampal volumes after CSE

The mean interrater CoV for hippocampal volumetry was 6.27% and intrarater CoV was 5.19% (MY)/7.88% (MM) for each observer, respectively. These values are consistent with reported values from a sample of published studies (Jack et al., 1990; Obenaus et al., 2001; Gousias et al., 2008) and were considered adequate to proceed with further analysis.

On cross-sectional analysis after correction for ICV, children with CSE did not have significantly different hippocampal volumes from controls at any time point (ANOVA, p > 0.1; Table 3).

**Table 3:** Mean adjusted hippocampal volumes at 1, 6, and 12 months post-CSE

		Mean hippocampal volumes (mm^3^) (95% CI) after adjustment for ICV		
Time point	Side	PFS	Non-PFS	Controls
1 month	Left	1,942 (1,856–2,028)	1,862 (1,790–1,934)	1,975 (1,886–2,064)
	Right	2,046 (1,952–2,140)	1,943 (1,864–2,021)	2,083 (1,986–2,180)
6 months	Left	1,980 (1,870–2,088)	1,911 (1,819–2,004)	2,062 (1,972–2,152)
	Right	2,167 (2,039–2,295)	1,930 (1,822–2,039)*	2,175 (2,070–2,281)
12 months	Left	2,052 (1,939–2,165)	1,895 (1,796–1,994)*	2,077 (1,984–2,170)
	Right	2,202 (2,053–2,351)	1,994 (1,864–2,125)	2,188 (2,065–2,310)

* Significantly different from control values p < 0.05.

#### Hippocampal growth over time

**Figure 1:**
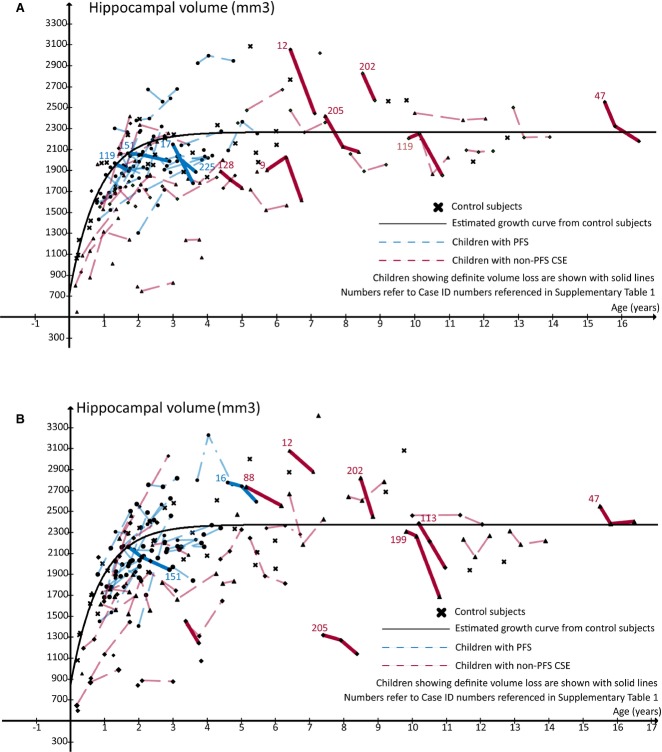
Left (A) and right (B) hippocampal growth in the year following CSE. Children showing definite hippocampal volume loss are shown with solid lines.

Forty-nine children attended the second assessment, and a further 11 children attended a third scan (but not the second); therefore, 60/80 children underwent at least two assessments and were able to have hippocampal growth rates estimated. Fifteen 60 (25%, 95% confidence interval [CI] 15.7–37.2%) showed definite decreases in hippocampal volume. They were split between PFS (5/26) and non-PFS (10/34) groups as shown in Table 4. A summary of the clinical details of each patient with volume loss is available online (Table S1). The mean reduction in hippocampal volume found was 398 mm^3^ (standard deviation [SD] 261 mm^3^), representing 17% of initial hippocampal volume. 4/5 of the children with PFS showed unilateral loss, whereas only 5/10 of those with non-PFS CSE did. Figure 1 shows left and right hippocampal volumes over time for the entire cohort, with those patients showing a definite decrease highlighted with solid lines.

**Table 4:** Proportion of children showing definite hippocampal volume loss

	No. children with two or more MRI scans	No. showing definite volume loss (%)
Prolonged febrile seizures	26	5 (19.2)
Non-PFS CSE	34	10 (29.4)
Total	60	15 (25.0)

### Predictive factors for hippocampal volume loss

There was no statistically significant difference in the proportion of children with definite volume loss in PFS/non-PFS groups (p = 0.26, chi-square).

Linear regression did not show any significant association between hippocampal growth rates (left/right) and a number of clinical factors, including the following: seizure duration (p = 0.150/0.425); focal versus generalized CSE (p = 0.253/0.124); continuous versus intermittent CSE (p = 0.941/0.742); history of CSE (p = 0.150/0.096); or number of previous seizures (p = 0.948/0.541). Each factor was entered individually into the regression model (p-values quoted) and in all two-factor combinations, with no significant associations found. Logistic regression was also performed using a definite decrease in hippocampal volume as the outcome, and the same predictors and did not show any significant associations, although there was a borderline association between volume loss and a history of previous seizures (p = 0.063, odds ratio [OR] 3.56; 95% CI 0.93–13.8). Older children were more likely to show volume loss (p = 0.003, OR 1.31; 95% CI 1.01–1.55); however, this is confounded by the fact that younger children would be expected to be growing at a fast rate, and therefore, less likely to show volume loss, even if growth failure did occur.

Regression analysis on the PFS group alone showed that the number of previous febrile seizures had a significant negative association with left hippocampal growth (p < 0.001; B = −76.68; 95% CI −111.16 to −42.20) and that children who showed a definite decrease in hippocampal volume were more likely to have had previous febrile seizures (p = 0.051, OR 1.75; 95% CI 1.00–3.06). No associations were found with other clinical factors.

Thirty-one of 80 children experienced seizure recurrence during the follow-up period, of which 6 had a further episode of CSE. Recurrent seizures were not associated with a definite decrease in hippocampal volume, but there was a borderline association between hippocampal volume loss and repeated CSE (p = 0.079, OR 4.61; 95% CI 0.84–25.5).

## Discussion

To our knowledge this is the first study to show that hippocampal volume loss occurs in a significant proportion of children following all types of CSE and not only in those who have PFS, as most previous studies have not included children with etiologies other than PFS. This is important because although attention has focused on PFS as a risk factor for MTS, it suggests that similar consideration needs to be paid to the possibility of hippocampal damage in other forms of CSE.

Although previous observations did not distinguish between different causes of CSE (Meyer et al., 1954), later studies have suggested a specific relationship between MTS and childhood PFS (French et al., 1993; Kuks et al., 1993), although associations with other neurologic insults such as bacterial meningitis have also been reported (Harvey et al., 1995).

If childhood CSE directly causes the development of MTS, progressive volume loss should be a necessary step in the transformation of a normal hippocampus into a sclerosed and epileptogenic one. Although it cannot be assumed that all children showing early volume loss will progress further to clinical MTS, it seems biologically implausible that those who are not showing signs of altered hippocampal growth will go on to develop MTS as a result of this episode of CSE if no signs of this process are detectable in the first year. Therefore, around 25% of children with CSE are likely to be at risk of developing MTS, but further study is required to determine how many of these children will ultimately develop TLE.

Given the finding that hippocampal volume loss occurs in similar, if not greater frequency after non-PFS CSE, it is unclear why this is not also associated with MTS/TLE. One possibility is that MTS is underdiagnosed in patients with non-PFS CSE. Diagnosing MTS requires specific MRI sequences that are optimized to detect hippocampal pathology, which may not have been available in historical cases (Geuze et al., 2005). Furthermore, MRI may not always be performed in these patients as patients with idiopathic epilepsy or a known symptomatic cause for their seizures are unlikely to have repeated MRI unless there is an unexpected clinical deterioration, especially given the challenges of performing this in children with a high burden of neurodisability.

Alternatively differences in the pathology of the underlying conditions may mean that, although the initial hippocampal injury is similar in all forms of CSE, the longer term course differs. Although studies of adults with chronic epilepsy have shown that between 10% and 20% show hippocampal volume loss on longitudinal MRI (Liu et al., 2005; Salmenperä et al., 2005) that does not appear to be associated with MTS, data from animal studies suggest that preexisting neuronal abnormalities or insults are likely to increase rather than decrease the vulnerability of the hippocampus to further injury (Jensen et al., 1992; Lucas et al., 2011).

Only two other studies to date have reported longitudinal measurements of hippocampal volumes after CSE in children (Scott et al., 2003; Provenzale et al., 2008). In both these studies, which only considered children with PFS, initial hippocampal volumes were measured within 3–5 days of the PFS, with follow-up measurements months to years later. It is known that patients imaged within this acute period show increased hippocampal volumes, thought to be attributable to hippocampal edema (Scott et al., 2006). Scott et al. (2003) interpreted a fall of 203 mm^3^ in mean corrected hippocampal volumes in children with PFS between initial MRI scan and follow-up at 4–8 months post-CSE as the resolution of this edema, since hippocampal volumes at follow-up were not significantly different from control values. Provenzale et al. (2008) took initial measurements at a similar time point, but had a longer period of follow-up spanning 2–23 months post-CSE. They reported absolute falls of 715–1,217 mm^3^ in uncorrected hippocampal volumes in 3/11 children along with persistent abnormal T_2_ signals, and smaller reductions in a further two children. Because confounding by the hippocampal edema present in the acute phase makes it difficult to interpret subsequent falls in hippocampal volume, this study was designed so that the initial measurements were made at a point when this would be expected to have resolved. That the results from our study are broadly in line with these previous studies, demonstrates that the effect on hippocampal growth does not simply represent resolution of the edema. It is important to note that several children with definite volume loss had final hippocampal volumes that lie within the normal range. Therefore, cross-sectional studies are less likely to reveal evidence for hippocampal abnormalities. This highlights the importance of carrying out longitudinal studies to address the issue of hippocampal injury following CSE.

The initial MRI findings in this cohort have been reported previously (Yoong et al., 2012); one patient had preexisting MTS and one patient met criteria for unilateral hippocampal malrotation. None of the children with PFS had hippocampal or extrahippocampal abnormalities on their initial MRI, and none has developed unprovoked seizures or MTS to date, although some have had recurrent febrile seizures. This loss of volume in a significant proportion of children appears to represent a new and progressive hippocampal injury: a potential precursor to MTS over the longer term.

### Limitations

There are a number of limitations that need to be addressed when interpreting the results of this study. First, as previously mentioned, direct comparison of growth rates between patients and controls was not possible. Studies of hippocampal growth in healthy children (Pfluger et al., 1999; Utsunomiya et al., 1999; Gogtay et al., 2006; Knickmeyer et al., 2008) have shown that the normal hippocampus increases rapidly in size over the first 2 years of life and more slowly thereafter; therefore, it seems reasonable to conclude that a loss of hippocampal volume is pathologic at this age. Our definition of “definite” volume loss is a conservative one, as growth failure may exist at this age range without actual loss of volume.

Second, few children with acute symptomatic CSE took part in the study and only one was able to undergo repeat MRI. This was because a disproportionate number of patients with acute symptomatic CSE declined consent or were not able to take part due to the severity of their clinical condition. As a result, the non-PFS group consists predominantly of children with prior neurologic injuries and genetic/idiopathic epilepsy. This necessarily limits the conclusions that can be drawn about children with acute injuries.

Third, the use of linear regression to estimate growth rates assumes that growth can be approximated by a linear function. Using a regression technique, with multiple measurements by multiple observers reduces the effects of any natural variability in hippocampal measurements, thereby minimizing the risk of type 1 error, but means that children whose hippocampus grows initially and only starts to lose volume after 6 months may not have been detected. Although we believe an immediate effect on growth is more likely, it is not impossible that an unknown delayed pathologic process is important in the pathogenesis of MTS.

Despite these limitations, our study is the largest study of childhood CSE of different etiologies and with longitudinal follow-up at three time points within a year of initial CSE. The lack of association between hippocampal growth and any seizure-associated variables, suggests that there may not be a simple relationship between seizure severity and hippocampal damage.

Despite previous studies that suggest an association between seizure duration and MTS (Maher & McLachlan, 1995), we did not find any association between CSE duration and hippocampal volume loss, albeit all the children in our cohort had seizures >30 min. We did find an association between volume loss and previous febrile seizures, as well as borderline associations with any previous seizures and subsequent episodes of CSE, suggesting that “multiple hits” may be required to cause lasting hippocampal damage and that the number of significant events may be important in the development of MTS. The lack of a significant dose–response effect may be due to several patients who did not show volume loss despite having recurrent seizures on a weekly or daily basis, but further data are needed to draw a definite conclusion.

## Conclusion

We have demonstrated that approximately 20–30% of children have definite hippocampal volume loss in the year following CSE. In comparison to previous studies of hippocampal volume after CSE, we have achieved a more intensive and more complete follow-up and included etiologies of CSE other than PFS. This has shown that volume loss after CSE is not limited to PFS, but occurs with equal, if not greater frequency, in other forms of CSE.

There may be a role of repeated febrile seizures or repeated episodes of CSE in the pathogenesis of long-term hippocampal injury, but there does not appear to be a strong association with any other clinical factors. Further identification of specific risk factors for hippocampal volume loss may be possible with larger cohorts of children with CSE, such as the FEBSTAT study (Hesdorffer et al., 2012), but for the present remains a challenge. It is apparent that there is a risk of progressive hippocampal damage following CSE and that this can occur after CSE of any etiology. Longer term follow-up of this and other cohorts will be required to determine the risks of this initial hippocampal volume loss further progressing to clinical MTS, as well as clarifying the association or lack thereof with non-PFS CSE.

## Supporting Information

**Table S1.** Details of children showing hippocampal volume loss following status epilepticus.

## References

[b1] Cascino GD (1995). Clinical correlations with hippocampal atrophy. Magn Reson Imaging.

[b2] Cavanagh JB, Meyer A (1956). Aetiological aspects of Ammon's horn sclerosis associated with temporal lobe epilepsy. BMJ.

[b3] Chin RFM, Neville BGR, Peckham C, Bedford H, Wade A, Scott RC (2006). Incidence, cause, and short-term outcome of convulsive status epilepticus in childhood: prospective population-based study. Lancet.

[b4] Farrow TFD, Dickson JM, Grünewald RA (2006). A six-year follow-up MRI study of complicated early childhood convulsion. Pediatr Neurol.

[b5] French JA, Williamson PD, Thadani VM, Darcey TM, Mattson RH, Spencer SS, Spencer DD (1993). Characteristics of medial temporal lobe epilepsy: I. Results of history and physical examination. Ann Neurol.

[b6] Geuze E, Vermetten E, Bremner JD (2005). MR-based in vivo hippocampal volumetrics: 1. Review of methodologies currently employed. Mol Psychiatry.

[b7] Gogtay N, Nugent TF, Herman DH, Ordonez A, Greenstein D, Hayashi KM, Clasen L, Toga AW, Giedd JN, Rapoport JL, Thompson PM (2006). Dynamic mapping of normal human hippocampal development. Hippocampus.

[b8] Gousias IS, Rueckert D, Heckemann RA, Dyet LE, Boardman JP, Edwards AD, Hammers A (2008). Automatic segmentation of brain MRIs of 2-year-olds into 83 regions of interest. NeuroImage.

[b9] Grattan-Smith JD, Harvey AS, Desmond PM, Chow CW (1993). Hippocampal sclerosis in children with intractable temporal lobe epilepsy: detection with MR imaging. AJR Am J Roentgenol.

[b10] Hammers A, Allom R, Koepp MJ, Free SL, Myers R, Lemieux L, Mitchell TN, Brooks DJ, Duncan JS (2003). Three-dimensional maximum probability atlas of the human brain, with particular reference to the temporal lobe. Hum Brain Mapp.

[b11] Harvey AS, Grattan-Smith JD, Desmond PM, Chow CW, Berkovic SF (1995). Febrile seizures and hippocampal sclerosis: frequent and related findings in intractable temporal lobe epilepsy of childhood. Pediatr Neurol.

[b12] Hesdorffer DC, Shinnar S, Lewis DV, Moshé SL, Nordli DR, Pellock JM, Macfall J, Shinnar RC, Masur D, Frank LM, Epstein LG, Litherland C, Seinfeld S, Bello JA, Chan S, Bagiella E, Sun S, the FEBSTAT study team (2012). Design and phenomenology of the FEBSTAT study. Epilepsia.

[b13] Jack R, Bentley D, Twomey K, Zinsmeister R (1990). MR imaging-based volume measurements of the hippocampal formation and anterior temporal lobe: validation studies. Neuroradiology.

[b14] Jensen FEE, Holmes GLL, Lombroso CTT, Blume HKK, Firkusny IRR (1992). Age-dependent changes in long-term seizure susceptibility and behavior after hypoxia in rats. Epilepsia.

[b15] Knickmeyer RC, Gouttard S, Kang C, Evans D, Wilber K, Smith JK, Hamer RM, Lin W, Gerig G, Gilmore JH (2008). A structural MRI study of human brain development from birth to 2 years. J Neurosci.

[b16] Kuks JBM, Cook MJ, Fish DR, Stevens JM, Shorvon SD (1993). Hippocampal sclerosis in epilepsy and childhood febrile seizures. Lancet.

[b17] Liu RSN, Lemieux L, Bell GS, Sisodiya SM, Bartlett PA, Shorvon SD, Sander JWAS, Duncan JS (2005). Cerebral damage in epilepsy: a population-based longitudinal quantitative MRI study. Epilepsia.

[b18] Lucas MM, Lenck-Santini P-P, Holmes GL, Scott RC (2011). Impaired cognition in rats with cortical dysplasia: additional impact of early-life seizures. Brain.

[b19] Maher J, McLachlan RS (1995). Febrile convulsions. Is seizure duration the most important predictor of temporal lobe epilepsy?. Brain.

[b20] Meyer A, Falconer MA, Beck E (1954). Pathological findings in temporal lobe epilepsy. J Neurol Neurosurg Psychiatry.

[b21] Natsume J, Bernasconi N, Miyauchi M, Naiki M, Yokotsuka T, Sofue A, Bernasconi A (2007). Hippocampal volumes and diffusion-weighted image findings in children with prolonged febrile seizures. Acta Neurol Scand Suppl.

[b22] Obenaus A, Yong-Hing CJ, Tong KA, Sarty GE (2001). A reliable method for measurement and normalization of pediatric hippocampal volumes. Pediatr Res.

[b23] Pfluger T, Weil S, Weis S, Vollmar C, Heiss D, Egger J, Scheck R, Hahn K (1999). Normative volumetric data of the developing hippocampus in children based on magnetic resonance imaging. Epilepsia.

[b24] Pohlmann-Eden B (2004). Evolution of MRI changes and development of bilateral hippocampal sclerosis during long lasting generalised status epilepticus. J Neurol Neurosurg Psychiatry.

[b25] Provenzale JM, Barboriak DP, VanLandingham K, MacFall J, Delong D, Lewis DV (2008). Hippocampal MRI signal hyperintensity after febrile status epilepticus is predictive of subsequent mesial temporal sclerosis. AJR Am J Roentgenol.

[b26] Salmenperä T, Könönen M, Roberts N, Vanninen R, Pitkänen A, Kälviäinen R (2005). Hippocampal damage in newly diagnosed focal epilepsy: a prospective MRI study. Neurology.

[b27] Scott RC, Gadian DG, King MD, Chong WK, Cox TC, Neville BGR, Connelly A (2002). Magnetic resonance imaging findings within 5 days of status epilepticus in childhood. Brain.

[b28] Scott RC, King MD, Gadian DG, Neville BGR, Connelly A (2003). Hippocampal abnormalities after prolonged febrile convulsion: a longitudinal MRI study. Brain.

[b29] Scott RC, King MD, Gadian DG, Neville BGR, Connelly A (2006). Prolonged febrile seizures are associated with hippocampal vasogenic edema and developmental changes. Epilepsia.

[b30] Shinnar S, Bello JA, Chan S, Hesdorffer DC, Lewis DV, Macfall J, Pellock JM (2012). MRI abnormalities following febrile status epilepticus in children: the FEBSTAT study. Neurology.

[b31] Tarkka R, Pääkkö E, Pyhtinen J, Uhari M, Rantala H (2003). Febrile seizures and mesial temporal sclerosis: no association in a long-term follow-up study. Neurology.

[b32] Utsunomiya H, Takano K, Okazaki M, Mitsudome A (1999). Development of the temporal lobe in infants and children: analysis by MR-based volumetry. AJNR Am J Neuroradiol.

[b33] VanLandingham KE, Heinz ER, Cavazos JE, Lewis DV (1998). Magnetic resonance imaging evidence of hippocampal injury after prolonged focal febrile convulsions. Ann Neurol.

[b34] Yoong M, Madari R, Martinos M, Clark C, Chong K, Neville B, Chin R, Scott R (2012). The role of magnetic resonance imaging in the follow-up of children with convulsive status epilepticus. Dev Med Child Neurol.

