# Supplementary material for: Hippocampal volume loss following childhood convulsive status epilepticus is not limited to prolonged febrile seizures
Source: Epilepsia. 2013 Oct 28;54(12):2108–15. doi: 10.1111/epi.12426 (PMC4377099; doi:10.1111/epi.12426)
Supplement: Supplementary file 1 — Table S1. Details of children showing hippocampal volume loss following status epilepticus. [file epi0054-2108-sd1.docx]

| Patient ID | Aetiology | Age at CSE (years) | Duration (min) | Previous seizure history | Focal | Continuous | Clinical information | | Medication | Seizure recurrence | Hippocampal changes | Initial hippocampal volume (mm^3^) | | Laterality of volume loss |
| --- | --- | --- | --- | --- | --- | --- | --- | --- | --- | --- | --- | --- | --- | --- |
|  |  |  |  |  |  |  |  |  |  |  |  | Left | Right |  |
| 16 | PFS | 4.56 | 69 | No previous seizures | No | No | PFS | | None | No further seizures | Decrease in right hippocampal volume: 240mm^3^/yr | 2089 | 2774 | Unilateral |
| 17 | PFS | 2.96 | 45 | 2 previous febrile convulsions, 1 episode PFS | No | Yes | PFS | | None | 2 further PFS | Decrease in left hippocampal volume: 215mm^3^/yr | 2034 | 2225 | Unilateral |
| 119 | PFS | 1.21 | 60 | 2 previous febrile convulsions from 6 months age | No | Yes | PFS | | None | 1 further short febrile convulsion | Decrease in left hippocampal volume: 197mm^3^/yr | 1955 | 1889 | Unilateral |
| 151 | PFS | 1.61 | 105 | No previous seizures | No | Yes | PFS | | None | No further seizures | Small bilateral decreases in hippocampal volume: left 67 mm^3^/yr; right 178 mm^3^/yr | 2063 | 2162 | Bilateral |
| 225 | PFS | 2.89 | 30 | 2 previous febrile convulsions | No | No | PFS | | None | No further seizures | Decrease in left hippocampal volume: 660mm^3^/yr | 2147 | 1968 | Unilateral |
| 9 | Non-PFS | 5.68 | 30 | 6 previous short febrile seizures | No | No | Ex-prem 26/40  Previous IVH  Developmental delay | | None | No further seizures | Decrease in left hippocampal volume 292mm^3^/yr | 1903 | 2342 | Unilateral |
| 12 | Non-PFS | 6.34 | 45 | 1 previous unprovoked focal seizure | Yes | No | Idiopathic epilepsy | | None | Recurrent episode of CSE | Decrease in left hippocampal volume 860mm^3^/yr, right 286mm^3^/yr | 3056 | 3080 | Bilateral |
| 47 | Non-PFS | 15.44 | 103 | Daily seizures for past 1 ½ years | No | No | Cryptogenic epilepsy | | Sodium valproate  Topiramate | Daily recurrent seizures, myoclonic and generalised tonic-clonic | Decrease in left hippocampal volume 360mm^3^/yr, right 130mm^3^/yr | 2555 | 2551 | Bilateral |
| 88 | Non-PFS | 5.10 | 30 | Previous PFS followed by 2 subsequent simple febrile convulsions | No | Yes | Idiopathic epilepsy | | Phenytoin  Carbamazepine | No further seizures | Decrease in right hippocampal volume 174mm^3^/yr | 2476 | 2733 | Unilateral |
| 113 | Non-PFS | 10.10 | 215 | 3 neonatal seizures | No | Yes | Ex-prem, 24/40  Previous IVH  Developmental delay | | Carbamazepine | Started on medication, no further seizures | Decrease in right hippocampal volume 550mm^3^/yr | 2206 | 2387 | Unilateral |
| 128 | Non-PFS | 4.27 | 110 | Absence seizures since 6 months age, 10 prior episodes of CSE | No | No | Known developmental delay, seizures with acute febrile illness | | Topiramate | 5 absence seizures | Decrease in left hippocampal volume 252mm^3^/yr | 1891 | 2047 | Unilateral |
| 175 | Non-PFS | 3.31 | 45 | Neonatal seizures and then 5 seizures over past 3 months | No | No | Left occipital infarction from neonatal sepsis and haemorrhage | | Phenytoin,  Sodium Valproate | Weekly short clonic seizures | Decrease in right hippocampal volume 544mm^3^/yr | 1233 | 1455 | Unilateral |
| 199 | Non-PFS | 9.83 | 47 | None | Yes | Yes | Unprovoked new onset CSE | | None | 1 further possible seizure | Bilateral decrease in hippocampal volume: left 410mm^3^/yr; right 685mm^3^/yr | 2207 | 2309 | Bilateral |
| 202 | Non-PFS | 8 | 90 | 1 previous febrile convulsion, previous cluster of clonic seizures | Yes | No | Cryptogenic epilepsy | | Sodium valproate | None | Bilateral decreases in hippocampal volume: left 442mm^3^/yr; right 1017mm^3^/yr | 2828 | 2818 | Bilateral |
| 205 | Non-PFS | 7.36 | 30 | 8 previous CPS | Yes | Yes | Right mesial temporal sclerosis | | None | Monthly CPS | Bilateral decreases in hippocampal volume: left 360mm^3^/yr; right 185mm^3^/yr | 2422 | 1321 | Bilateral |
| CPS: Complex partial seizures  IVH: Intraventricular haemorrhage  PFS: Prolonged Febrile Seizure | | | | | | | | Table 5: Details of children showing hippocampal volume loss following status epilepticus | | | | | | |
