# Supplementary material for: Hippocampal volume loss following childhood convulsive status epilepticus is not limited to prolonged febrile seizures
Source: Epilepsia. 2013 Oct 28;54(12):2108–15. doi: 10.1111/epi.12426 (PMC4377099; doi:10.1111/epi.12426)
Supplement: Supplementary file 2 — Table S1. Details of children showing hippocampal volume loss following status epilepticus. [file epi0054-2108-sd2.pptx]

## Slide 1
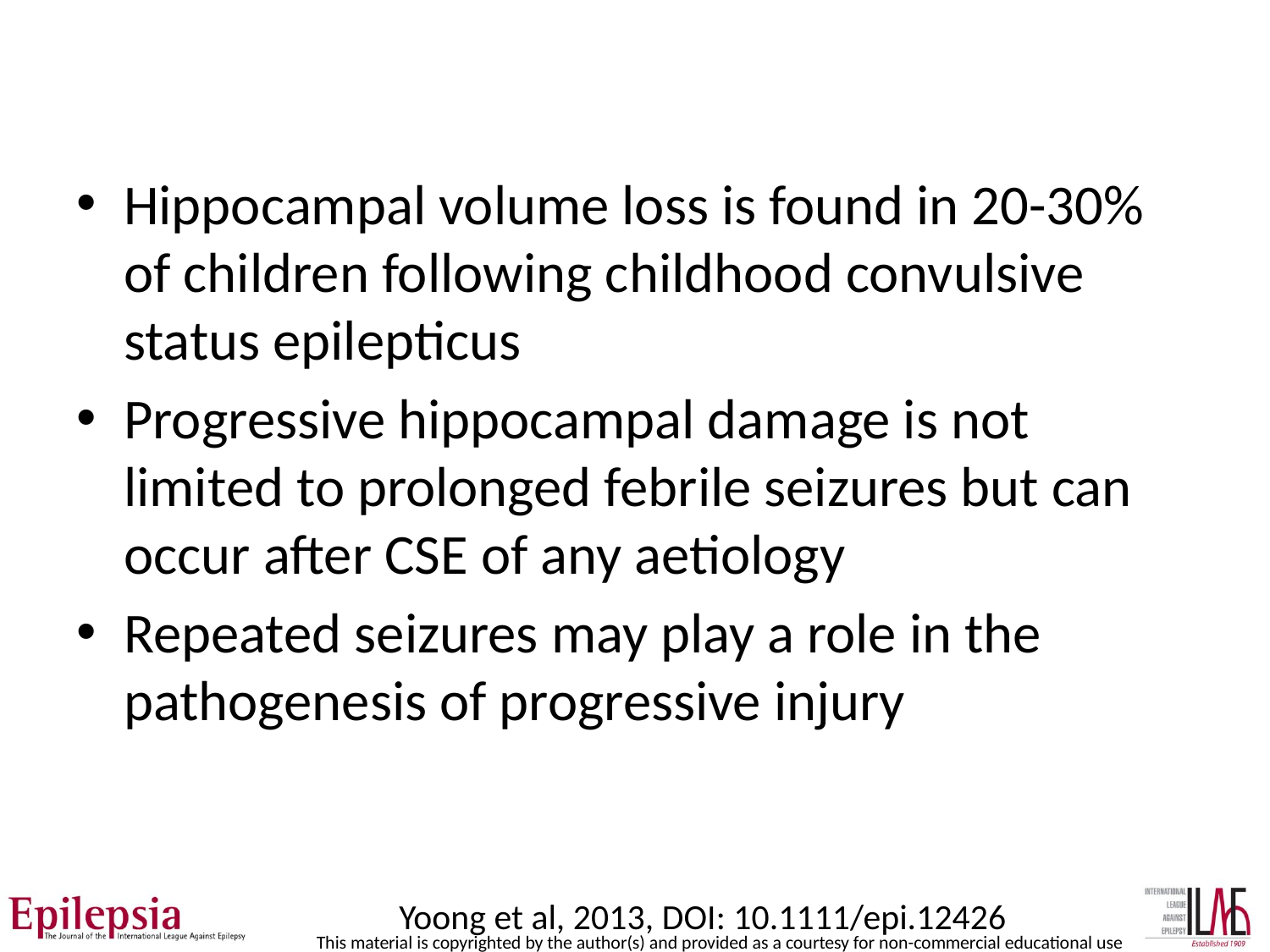

Hippocampal volume loss is found in 20-30% of children following childhood convulsive status epilepticus
Progressive hippocampal damage is not limited to prolonged febrile seizures but can occur after CSE of any aetiology
Repeated seizures may play a role in the pathogenesis of progressive injury
Yoong et al, 2013, DOI: 10.1111/epi.12426
This material is copyrighted by the author(s) and provided as a courtesy for non-commercial educational use
